# Safety and feasibility of a novel multielectrode array catheter in mapping atrial and ventricular arrhythmias with high density: results from the multicenter OPTIMUM study

**DOI:** 10.1007/s10840-023-01568-y

**Published:** 2023-05-25

**Authors:** Johan Vijgen, Gediminas Račkauskas, Dagmara Dilling-Boer, Andrea Sarkozy, Giuseppe Ciconte, Gabriele Giuseppe Vicedomini, Carlo Pappone

**Affiliations:** 1 Jessa Ziekenhuis, Campus Virga Jesse, Hasselt, Belgium; 2 Vilnius University Hospital Santaros Clinic, Vilnius, Lithuania; 3 Cardiology Department, University Hospital of Antwerp, Antwerp, Belgium; 4 University of Antwerp, Antwerp, Belgium; 5 IRCCS Policlinico San Donato, Milan, Italy

The OPTRELL (Biosense Webster) multielectrode array mapping catheter includes 48 electrodes symmetrically distributed on 6 splines. A unique feature is an additional unipolar reference electrode (TRUERef, Biosense Webster) located in the distal end of the irrigation lumen, serving as an internal close unipolar reference electrode to reduce far-field unipolar signals. The catheter provides visualization of real-time conduction vectors for better understanding of beat-by-beat wave propagation over the catheter surface, showing the direction of most significant change in local activation times and highlighting areas of slow conduction and scarring. An enhanced bipolar mapping module enables selection of the measurement either along or across the splines, whichever best conforms with wave propagation. Studies in a swine model demonstrated the ability of this catheter to provide high-density and high-resolution mapping in complex substrates [1, 2]. We aimed to assess the safety and mapping performance of the catheter and navigation system for mapping the atria and ventricles in patients with various complex arrhythmias.

The OPTIMUM study (ClinicalTrials.gov Identifier: NCT04983797) was a prospective, a multicenter, a single-arm, and an open-label study. Participants with scar-related atrial tachycardia (AT), paroxysmal or persistent atrial fibrillation (PAF or PsAF), ventricular tachycardia (VT), or premature ventricular complexes (PVCs) underwent preablation mapping with the study catheter (Fig. 1), followed by standard-of-care ablation. Primary safety endpoint was 7-day incidence of device-related serious adverse events (SAEs), and primary effectiveness endpoint was completion of protocol-required fast activation and electro anatomical preablation mapping without resort to nonstudy mapping catheter(s). Physicians’ assessment of deployment, maneuverability, and signal quality were obtained from a postprocedure survey using a 1‒7 Likert scale (1 = poor and 7 = excellent).

**Fig. 1:**
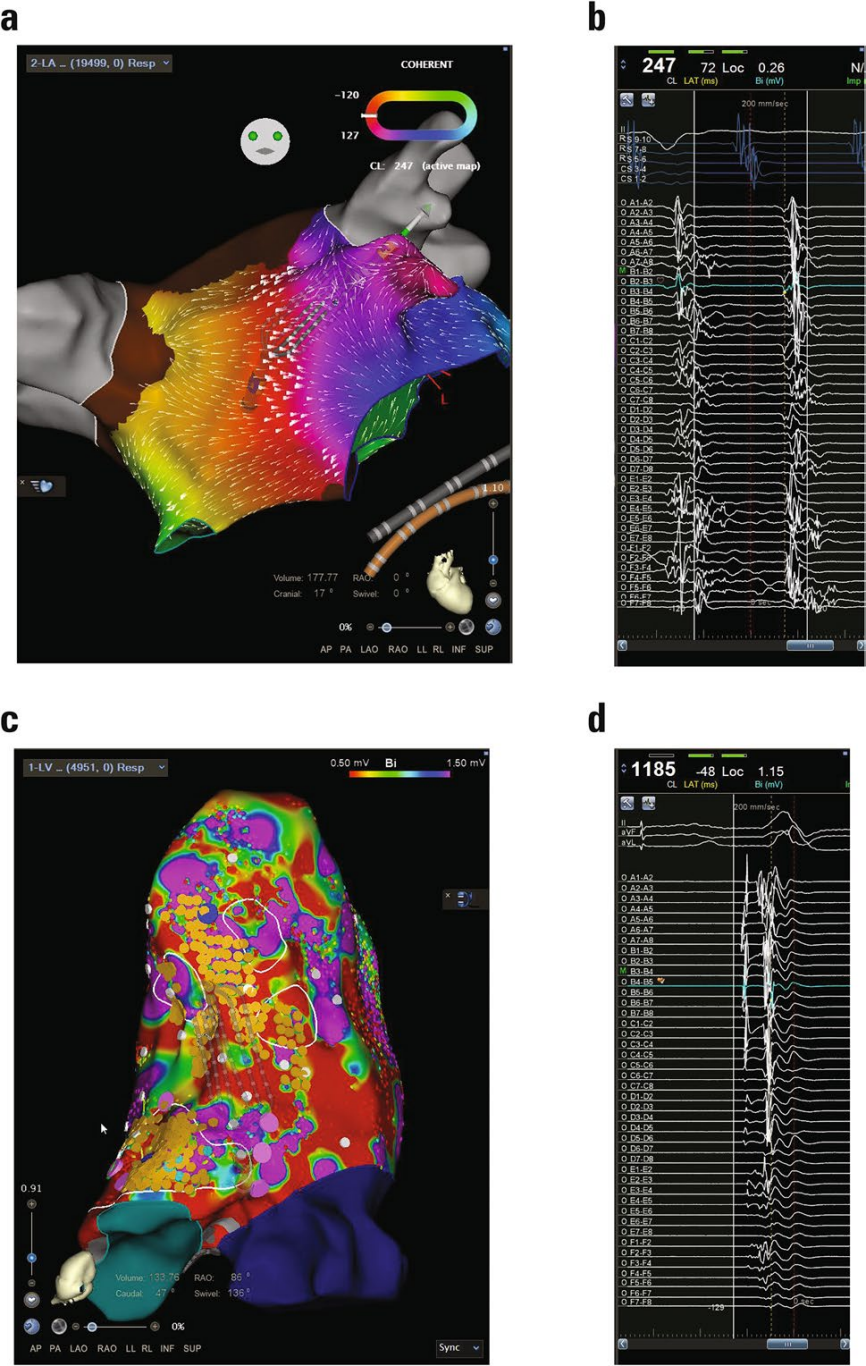
**a** High density mapping of an AT. The tachycardia was terminated by a linear ablation from the mitral valve to the superior right pulmonary vein through the scar zone. **b** The catheter is positioned on the anterior wall of the left atria and shows fractionated potentials and slow conduction in a scar zone. **c** Patient with dilated cardiomyopathy and intramural zones of delayed gadolinium enhancement inferior, inferoseptal, and inferolateral. Activation map with isochronal crowding and bipolar map with the position of the mapping catheter. **d** Recorded electrograms of the Purkinje fibers and ventricular myocardium. AT, atrial tachycardia

Between August 25 and October 26, 2021, 31 participants were enrolled and mapped (9 for scar-related AT, 12 for PAF, 1 for PsAF, 6 for VT, and 3 for PVC). Mean ± standard deviation (SD) age was 59.8 ± 14.4 years and 71% were male. Mean ± SD duration of preablation mapping was 29.9 ± 17.8, 20.0 ± 20.0, and 26.7 ± 8.8 min for AT, PsAF, and PAF, respectively, and 122.2 ± 43.9 and 51.3 ± 28.3 min for VT and PVC, respectively; mean total procedural time was 101.2 ± 32.5, 170.0, 102.5 ± 24.9, 223.7 ± 53.1, and 185.0 ± 25.5 min, respectively. Fast anatomical mapping was performed for all participants. Voltage mapping was performed in all redo-PAF, PsAF, and VT procedures and most AT and PVC procedures. Local activation-time mapping was most often performed for AT, VT, and PVC procedures.

No SAEs were related to the study catheter. Three participants reported 4 SAEs unrelated to the study catheter (pseudoaneurysm, hematoma, bladder catheter site injury, and anemia). All participants recovered or resolved at follow-up. There were no occurrences of cardiac perforation, thromboembolic events, catheter entanglement with cardiac structure, or device malfunction. Primary effectiveness endpoint was achieved in 100% of participants.

Almost all operators rated highly (≥ 5) on noise reduction in bipolar maps (30/31 operators) and short learning curve (27/31 operators). Operators rated the study catheter highly on: arrhythmogenicity versus PENTARAY (31/31 operators) and versus other multielectrode catheters (25/25 operators); design and coverage for confirming PVI versus PENTARAY (18/21 operators) and versus other multielectrode catheters (17/21 operators); and ability to characterize tissue versus PENTARAY (31/31 operators) and versus other multielectrode catheters (25/25 operators).

This first-in-human investigation of the novel multielectrode array mapping catheter provided clinical support for the results observed in preclinical studies. In a swine model, average voltage amplitudes between 2 sampling areas were comparable when using either standard or direction-aware bipolar mapping, suggesting minimal impact of bipolar map directionality. Minimizing directionality has the potential benefit of improving ability to recognize surviving myocardial bundles in the scar [2]; this was reflected in the physician feedback in our study, in which all 25 operators rated “ability to characterize tissue” above expectation compared with other multielectrode catheters.

In patients with complex cardiac arrhythmias, identification of the mechanisms maintaining or sustaining arrhythmia is a critical aspect of ablation procedures, and successful identification of these targets facilitates therapy [3]. The emergence of higher-resolution electroanatomic mapping with multielectrode catheters in recent years has been driven by this desire to better identify arrhythmia mechanisms and, from a more practical perspective, to reduce the mapping time required. The novel catheter may contribute to improved workflow and procedural efficiency with its high density, high resolution, and mapping efficiency with limited arrhythmogenicity. The “grid” design is also expected to facilitate understanding of conduction wavefront propagation and detailed mapping of complex circuits and mechanisms [1, 2]. Increased rapidity and coverage of mapping achieved with the novel catheter may confer benefit in cases of relative hemodynamic instability and in cases of unsustained arrhythmia. Higher-density mapping can be rapidly performed in regions of interest, including scar boundaries or near critical isthmuses.

Other grid-type catheters have recently been investigated in larger patient populations. Hsu et al. evaluated the EnSite Precision cardiac mapping system, which can be used with a variety of electrophysiology catheters; in that study, 101 of a total 925 procedures were performed with the Advisor HD Grid catheter [4]. The EnSite Precision system uses a hybrid magnetic and impedance-based catheter technology, incorporating surface electrodes, a separate reference, and a magnetic field frame. Fielder et al. evaluated electroanatomical mapping in 334 patients with PsAF using the Advisor HD Grid mapping catheter with EnSite Precision and HD Wave Solution electrode configuration to generate multiple map types in a variety of rhythms [5]. There are currently no published randomized trials comparing different mapping systems, and the current observational study was not set up to compare to other mapping systems. Our study population is not comparable to other published data since we focused on macro-reentrant AT and VT, while other studies focused on PsAF, PAF, and typical atrial flutter.

Limitations of this study include the small sample size of 31 participants split across several arrhythmia subgroups, specifically chosen to demonstrate the performance of the catheter in the atria and ventricles. The purpose of the study was to show the safety and feasibility of the novel multielectrode array catheter in both atrial and ventricular arrhythmias as a first step before comparing it with other catheters. Subsequent studies including larger populations, comparison with other mapping catheters, and long-term outcomes of ablation will determine the performance of this catheter and its abilities in standard clinical use.

## Data Availability

Johnson & Johnson Medical Devices Companies have an agreement with the Yale Open Data Access (YODA) Project to serve as the independent review panel for evaluation of requests for clinical study reports and patient-level data from investigators and physicians for scientific research that will advance medical knowledge and public health. Requests for access to the study data can be submitted through the YODA Project site at http://yoda.yale.ed.
